# An Adaptive Grid Generation Approach to Pipeline Leakage Rapid Localization Based on Time Reversal

**DOI:** 10.3390/s25061753

**Published:** 2025-03-12

**Authors:** Yu Wang, Haoyang Chen, Yang Yang, Haoyu Zhou, Guangmin Zhang, Bin Ren, Yufei Yuan

**Affiliations:** 1 The International School of Microelectronics, Dongguan University of Technology, Dongguan 523808, China; chnwangyu@dgut.edu.cn (Y.W.); chy_249110@dgut.edu.cn (H.C.); yangyang@dgut.edu.cn (Y.Y.); 13412960597@163.com (H.Z.); 2 State Key Laboratory of Electronic Thin Films and Integrated Devices, University of Electronic Science and Technology of China, Chengdu 611731, China; 3 Zhuhai Kuaxinwei Technology Co., Ltd., Zhuhai 519000, China; yufeiyuan_ccmt@163.com

**Keywords:** pipeline leakage, adaptive grid generation, time reversal, negative pressure wave

## Abstract

Gas pipeline leakage will result in casualties and property losses if not detected in time. Conventional leakage localization methods usually rely on dense grid distribution, leading to high computational costs. This study proposes a time-reversal-based adaptive grid generation approach to enhance computational efficiency in pipeline leakage localization. The method introduces a resolution adjustment parameter to optimize captured signals, allowing for adaptive grid concentration in leakage areas based on energy distribution. Based on this principle, three steps—including signal adjustment computation, adaptive grid generation computation, and conventional TR localization computation based on the adaptive grids—are introduced. Then, an experimental study is conducted on a 55.8 m PVC pipeline with piezoceramic transducers, capturing negative pressure wave signals from four leakage points. The results demonstrate that the proposed approach maintains comparable localization accuracy while reducing the number of grids and localization time to only 0.6% and 2.4% of those required by conventional uniform grid methods, respectively. The findings demonstrate that the proposed method offers a computationally efficient and accurate solution for real-time pipeline leakage monitoring.

## 1. Introduction

Structural Health Monitoring (SHM) [1,2,3,4] plays a vital role in detecting various forms of structural damage [5,6], such as cracks [7,8], corrosion [9,10], connection looseness [11,12,13], bond slip [14], and other damages [15,16], either on demand or in real time [17]. With the increasing importance of pipeline networks for transporting gases, liquids, and slurry-like fluids [18,19], there has been a growing focus on pipeline SHM. However, pipeline failures, e.g., ruptures or cracks [20], often caused by corrosion [20,21,22], construction defects, or external damage [23,24], continue to occur, leading to catastrophic leaks. These incidents highlight the urgent need for reliable early detection systems. Various pipeline leak localization techniques [25] have been proposed, including transient model methods, mass/volume balance approaches [26], acoustic technology [27], hybrid methods [28], dynamic pressure wave methods [29], and machine learning methods [30]. Despite their potential, these methods face challenges such as high computational costs [25] and large localization errors [31]. In contrast, the negative pressure wave (NPW) method has gained significant attention due to its simplicity and high accuracy, offering an effective solution for pipeline leak detection.

When a leakage occurs, a negative pressure wave (NPW) propagates in both directions from the leakage point along the pipeline. By retracing the NPW path, the location of the leakage can be identified. Many efforts have been made to improve the accuracy of the NPW-based localization method [32]. For instance, Hu et al. [33] proposed a data-driven method based on NPW that helps highlight changing signal characteristics and overcomes the shortcomings of unclear signal characteristics. In order to eliminate noise interference, Zhu et al. [34] developed a novel pipeline pressure signal denoising method based on variational mode decomposition (VMD) to process negative pressure wave signals. However, certain acoustic features remain difficult to detect using these methods. For example, the −3 dB bandwidth, which serves as a critical boundary between leakage and non-leakage points, is often submerged in the results obtained through the aforementioned approaches. Complementarily, Ing et al. proved that it is feasible to identify key acoustic characteristics, such as −3 dB width and maximum peak, by implementing time-reversal (TR) localization methods [35].

Time-reversal (TR)-based leak localization methods leverage the unique ability of TR to focus signals precisely at their source [35]. The concept of TR is grounded in spatial reciprocity and can be applied in two distinct ways. The first is physical time reversal, commonly used in fields requiring the physical focusing of wave energy at a target location, such as non-destructive evaluation and detection [36]. The second approach is computational time-reversal localization [37,38], where signals are computationally re-radiated into the area of interest within a simulation environment, rather than in a real medium. In the computational process, grids are created within the monitoring region, and signals at these grids are computed by either convolving the time-reversed signal with the channel impulse response in the time domain or multiplying the time-reversed signal by the transfer function in the frequency domain [39].

The TR localization method, while offering richer signal characteristics, incurs significant computational cost due to the necessity of a large number of grids to ensure accurate target positioning. For instance, Cai et al. localized a target within a 1 m × 1 m area using 1,000,000 grids of 1 mm × 1 mm in size [40]. Similarly, Shi and Nehorai adopted 4 million grids (1 mm × 1 mm) to locate an object in a 2 m × 2 m monitoring area [41]. Mukherjee et al. employed 6000 uniform grids (0.05 m × 0.08 m) to identify targets with a 0.02 m radius in a 6 m × 4 m area using a time-reversal mirror [42]. Liao et al. utilized 180,000 grids to localize targets within a monitoring area of 45 cm × 40 cm [43]. The excessive computational load and high time consumption make real-time pipeline leakage monitoring with the TR localization technique highly challenging. On the other hand, even though there are other localization methods, such as compressive TR [44,45] and machine learning-based localization techniques [46,47], which can provide low computation, they often involve iterative optimization or require extensive training data, and may face challenges in adapting to different pipeline conditions.

In this paper, an adaptive grid generation approach based on TR is developed to speed up the leakage localization. Firstly, the acquired NPW signals are adjusted in order to decrease the localization resolution. Then, the adjusted signals are back-propagated in the monitoring area. According to the signal energy distribution in the monitoring area in the low-resolution situation, the grids can be adaptively generated. Finally, the signal energy map of the adaptive grids is calculated and plotted using the conventional TR localization method. An experiment was conducted to investigate the performance of the proposed approach. In the experiment, four valves were assembled along a 55.8 m PVC pipe to work as manually controllable leakages. The results indicate that the adaptive grids perform similarly to uniform grids, but the number of adaptive grids needed is only 0.6% of that of the necessary uniform grids. In addition, the adaptive-grid-based TR localization method consumes merely 2.4% of the time of the conventional one. Therefore, the proposed adaptive grid generation approach based on TR significantly reduces the number of grids and the computational burden compared to conventional methods, while still maintaining high localization accuracy.

The rest of this paper is organized as follows: Firstly, the proposed scheme is synthetically presented in Section 2. The measurement setup and scenarios follow in Section 3. The adaptive grid generation based on the measured data is described in Section 4. Furthermore, the leak localization results based on the adaptive grids and uniform grids are respectively shown and analyzed in Section 5. Section 6 discusses the performance of the proposed method. Section 7 concludes the paper. The parameters and symbols of the main notations appearing in this paper are shown in Table 1.

## 2. Description of the Principle

For a pressurized pipeline, a leakage at $r_{L}$ will generate a negative pressure wave signal $s(r_{L},t)$ that propagates along the pipeline. Two Lead Zirconate Titanate (PZT) sensors are surface-bonded on the pipeline near both ends, and they will capture the NPW signal. The locations of the two sensors are represented as $r_{1}$ and $r_{2}$, respectively.

**Figure 1:**
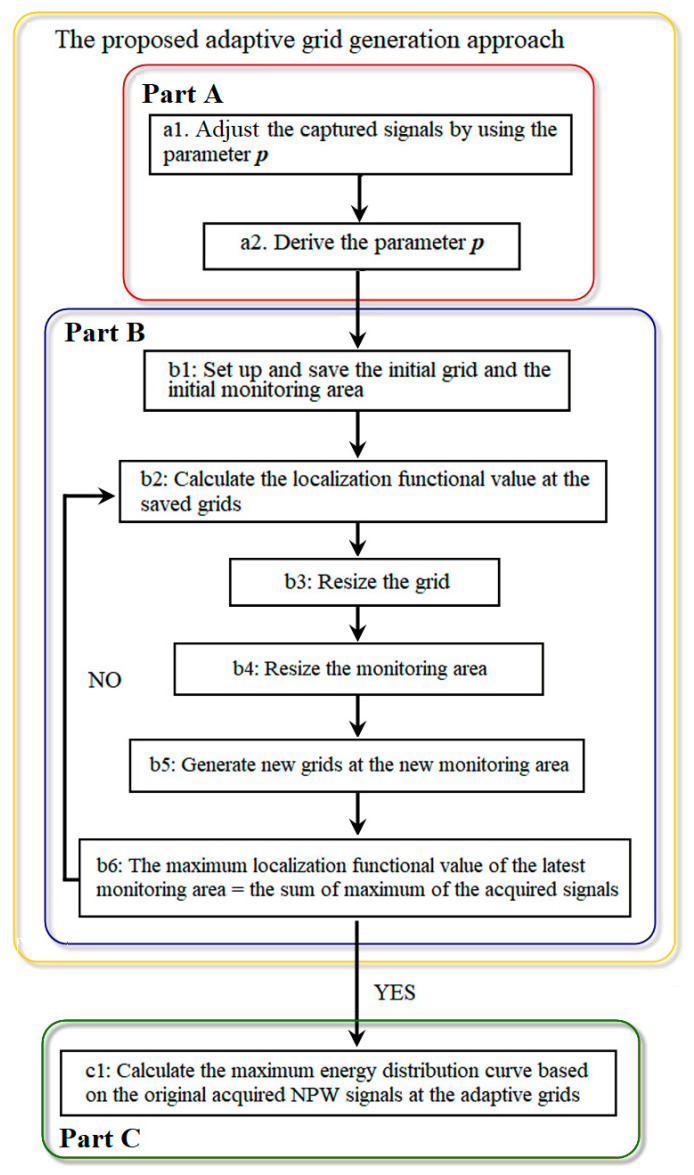
The flow diagram of the localization algorithm based on the adaptive grids.

In our work, the localization computation contains three steps, namely, the signal adjustment computation, the adaptive grid generation computation, and the conventional TR localization computation based on the adaptive grids, as shown in Figure 1. The steps are presented as follows:

### 2.1. The Signal Adjustment Computation

In the signal adjustment computation section, to avoid missing the leakage position due to using large grids, we should make the localization leakage area cover the entire monitoring region. In the proposed method, this is implemented by decreasing the localization resolution. Since the −3 dB value (0.7) sets a boundary limit between leakage points and non-leakage points, the localization functional value of the entire monitoring area should be larger than or equal to the −3 dB value of the sum of the maximum of the acquired signals. The detailed sub-steps are described as follows:

**a1.**  **Adjust the NPW signals captured by the two PZT sensors.**

The cross-correlation function between $x(r_{1},r_{L},t)$ captured by sensor 1 and $x(r_{2},r_{L},t)$ captured by sensor 2 is given as shown in Equation (1).

(1) $$x(r_{1},r_{L},t)⊗x(r_{2},r_{L},-t)=s(r_{L},t)⊗s(r_{L},-t)⊗a_{1,L,m}a_{2,L,m}\delta (t-t_{1,L,m}+t_{2,L,m})$$

where $t_{n,L,m}$ is the NPW propagation time from $r_{L}$ to $r_{n}$, and $\delta \left(t-t_{n,L,m}\right)$ and $a_{n,L,m}$ are the ideal impulse and the attenuation coefficient of the channel between $r_{L}$ and $r_{n}$, respectively. The subscript “m” corresponds to the forward propagation fields measured via the experiment. “⊗” represents the convolution operation.

Moreover, the self-correlation function of $x(r_{1},r_{L},t)$ is written as shown in Equation (2).

(2) $$x(r_{1},r_{L},t)⊗x(r_{1},r_{L},-t)=s(r_{L},t)⊗s(r_{L},-t)⊗a_{1,L,m}a_{1,L,m}\delta (t)$$

By using Fourier transform, compute $z_{1,L,2}(t)$ as shown in Equation (3).

(3) $$x(r_{1},r_{L},t)⊗x(r_{1},r_{L},-t)⊗z_{1,L,2}(t)=x(r_{1},r_{L},t)⊗x(r_{2},r_{L},-t)$$

Then, $z_{1,L,2}(t)$ can be represented as shown in Equation (4).

(4) $$z_{1,L,2}(t)=\frac{a_{2,L,m}}{a_{1,L,m}}\delta (t-t_{1,L,m}+t_{2,L,m})$$

Normalize the amplitude of $z_{1,L,2}(t)$ and enlarge the delay of $z_{1,L,2}(t)$ as follows in order to obtain $f_{12}(t)$, as shown in Equation (5):

(5) $$f_{12}(t)=\delta (t-p\times t_{1,L,m}+p\times t_{2,L,m})$$

where p is the resolution adjustment parameter (its physical meaning and determination method are described in detail in [48]).

The resolution adjustment parameter p is set as an unknown parameter. Using $f_{12}(t)$, we can adjust $x(r_{1},r_{L},t)$ and $x(r_{2},r_{L},t)$, as shown in Equations (6) and (7).

(6) $$x^{\prime }(r_{1},r_{L},t)=x(r_{1},r_{L},t)⊗f_{12}(-t)=e(r_{L},t)⊗a_{1,L,m}\delta (t+(p-1)\times t_{1,L,m}-p\times t_{2,L,m})$$

(7) $$x^{\prime }(r_{2},r_{L},t)=x(r_{2},r_{L},t)⊗f_{12}(t)=e(r_{L},t)⊗a_{2,L,m}\delta (t-p\times t_{1,L,m}+(p-1)\times t_{2,L,m})$$

**a2.**  **Derive parameter p.**

At a generic observation point $r_{k}$ of the monitoring area, the localization background functions of $x^{\prime }(r_{1},r_{L},t)$ and $x^{\prime }(r_{2},r_{L},t)$ are designated by Equations (8) and (9).

(8) $$h_{c}(r_{1},r_{k},t)=\delta (t+(p-1)\times t_{1,k,c}-p\times t_{2,k,c})$$

(9) $$h_{c}(r_{2},r_{k},t)=\delta (t+(p-1)\times t_{2,k,c}-p\times t_{1,k,c})$$

where $t_{1,k,c}$ is the NPW propagation time from $r_{k}$ to $r_{1}$ and $t_{2,k,c}$ is the NPW propagation time from $r_{k}$ to $r_{2}$. The subscript “c” represents that this corresponds to back-propagation fields, and can be obtained via calculations.

The adjusted signals are back-propagated, respectively, via Equation (10) [48].

(10) $$\left\{\begin{array}{c} g(r_{1},r_{k},t)=x^{\prime }(r_{1},r_{L},-t)⊗h_{c}(r_{1},r_{k},t)\\ g(r_{2},r_{k},t)=x^{\prime }(r_{2},r_{L},-t)⊗h_{c}(r_{2},r_{k},t) \end{array}\right.$$

In the proposed method, the moments corresponding to the −3 dB values of the signals $g(r_{1},r_{k},t)$ and $g(r_{2},r_{k},t)$ at the starting point and the end of the monitoring area are computed. To guarantee the localization leakage area covers the entire monitoring region, the −3 dB values of the signals $g(r_{1},r_{k},t)$ and $g(r_{2},r_{k},t)$ at the starting point and the end of the monitoring area should superpose each other. Thus, let

(11) $$\left\{\begin{array}{c} t_{1}^{bf}=t_{2}^{bs}\\ t_{1}^{es}=t_{2}^{ef} \end{array}\right.$$

where $t_{1}^{bf}$ is the moment corresponding to the first −3 dB value of the signal $g(r_{1},r_{k},t)$ at the starting point. $t_{2}^{bs}$ is the moment corresponding to the second −3 dB value of the signal $g(r_{2},r_{k},t)$ at the starting point. $t_{1}^{es}$ is the moment corresponding to the second −3 dB value of the signal $g(r_{1},r_{k},t)$ at the end. $t_{2}^{ef}$ is the moment corresponding to the first −3 dB value of the signal $g(r_{2},r_{k},t)$ at the end.

As illustrated in [48], the −3 dB width becomes narrow with the increase in parameter p. Thus, the minimum resolution adjustment parameter p, obtained by solving Equation (11), is set as the final resolution adjustment parameter p value, which will be utilized to generate the adaptive grids.

### 2.2. The Adaptive Grid Generation Computation

In this step, grids are concentrated in the leak area based on the energy distribution of the improved signals, which are adjusted by the specified parameter p. The detailed sub-steps are described as follows:

**b1**:  Set up the sizes of the initial grid and the initial monitoring area. Generate the initial grid and save their positions.
**b2**:  At the saved grid, calculate the localization functional value based on the parameter p determined by Equation (12) and ref. [48].
  (12) $$I_{o}(r_{k})=Max(\sum_{n=1}^{2}x^{\prime }(r_{n},r_{L},-t)⊗h_{c}(r_{n},r_{k},t))$$
**b3**:  Resize the grid to half of the previous grid size.
**b4**:  Resize the monitoring area. The center of the new monitoring area is the position of the maximum localization functional value obtained in step b2, and the range of the new monitoring area is set as the previous grid size.
**b5**:  Generate new grids at the new monitoring area by using the new grid size, and save the new grids’ positions.
**b6**:  Repeat step b2–step b5 until the maximum localization functional value of the latest monitoring area equals the sum of the maximum of the acquired signals.

It should be noted that steps b1–b6 correspond to Part B in Figure 1.

### 2.3. Conventional TR Localization Computation Based on the Adaptive Grids

In this step, the adaptive grids are applied to obtain the leak location as follows:

**c1**: Calculate and plot the maximum energy distribution curve according to the original acquired NPW signals by using the conventional TR localization method [35] at all the saved grids.
  (13) $$v_{o}(r_{k})=Max(\sum_{n=1}^{2}x(r_{n},r_{L},-t)⊗\delta (t-t_{n,k,c}))$$

It should be noted that although Equation (12) utilizes the adjusted signal energy distribution to adaptively mesh the monitoring area, Equation (13) employs the original NPW signals for localization, ensuring that the final leak point location remains accurate and consistent with the conventional TR methods, which will be demonstrated in Section 5.1.

It is worth mentioning that the proposed adaptive grid generation approach is composed of part A and B, as shown in Figure 1. In part A, the localization resolution is lowered by minimizing the resolution adjustment parameter p. In part B, the adaptive grids are generated based on the signal energy distribution in the low-resolution localization situation.

## 3. Experiment

**Figure 2:**
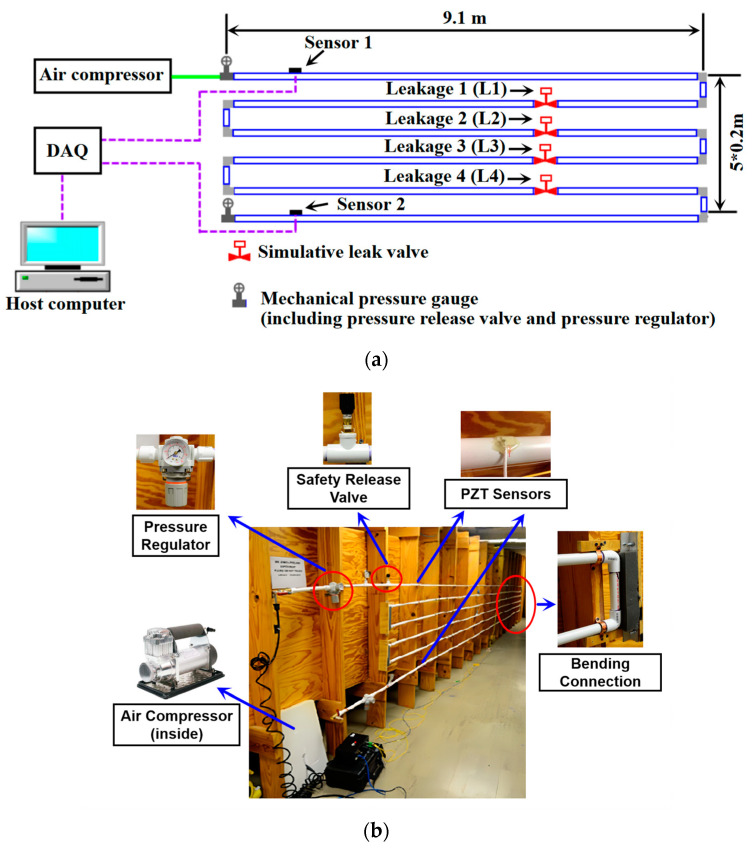
Schematic diagram and photo of the pipeline experiment. (**a**) Schematic diagram of the pipeline with PZT sensors. (**b**) Layout of the pipeline and setup details.

The full model pipeline is composed of a series of PVC pipe sections with a total length of 55.8 m, as shown in Figure 2. The pipeline has six 9.1 m straight sections connected by ten 90° elbow connectors and five 0.2 m sections. Regarding the pipeline, its wall thickness is 0.32 cm, and its diameter is 1.9 cm. Four manually controllable valves are utilized to serve as leakages and are, respectively, located 15.55 m, 24.84 m, 34.21 mm, and 43.47 m away from the inlet of the pipeline, as listed in Table 2. The outlet diameter of the valves is 6.35 mm. Since Lead Zirconate Titanate (PZT) material possesses a strong piezoelectric effect [49,50], energy harvesting ability [51,52], and wide bandwidth [53], PZT transducers are widely used as actuators to induce vibration and stress waves [54,55], as well as sensors to detect vibration and stress waves [56,57,58,59,60]. In our experiment, two PZT patch transducers with a size of 15 mm × 10 mm × 1 mm are bonded on the external surface of the pipeline to detect NPWs. The PZT transducers are, respectively, 1.32 m and 54.46 m away from the inlet, as listed in Table 3. The PZT material is APC850, and its parameters are listed in Table 4. It should be noted that as the lengths of the pipeline segments at the bends are too short to safely accommodate the installation of leakage valves, the locations of the leaks are all considered to be on straight pipes. However, as the sensors are placed at both ends of the pipeline, NPWs generated from leaks will propagate through the entire pipeline and be captured by both sensors, regardless of whether the leak occurs in a straight section or at a bend. Therefore, the proposed NPW method remains effective even if leaks occur at bends.

The data acquisition system is an NI PXI-5105 Digitizer (National Instruments, Austin, TX, USA) working in a DC software analog trigger mode with a signal trigger level of −0.02 V. The sampling rate is 100 KS/s. It should be noted that all PZT sensors and the data acquisition system were calibrated in accordance with the manufacturer’s instructions to guarantee the reliability of the experimental results.

A pressure regulator and safety release valve are installed for safety. In the experiment, a host computer equipped with i7-4702QM CPU, 16 GB DDR3 memory, and NVIDIA GT820M with 2 GB memory, is utilized for the localization computation.

The experiment was performed as follows: (1) The four valves were initially closed for pipeline pressurization with an air compressor. (2) In consideration of safety, the safety release valve was working to ensure the pipeline inner pressure was below 30 psi. For each test, the pipeline was pressurized up to 20 psi [61]. (3) An event of leakage was created through opening one of the valves on the pipeline. (4) The data acquisition system was triggered automatically by the NPW signal and recorded the signals captured by the PZT sensors.

**Figure 3:**
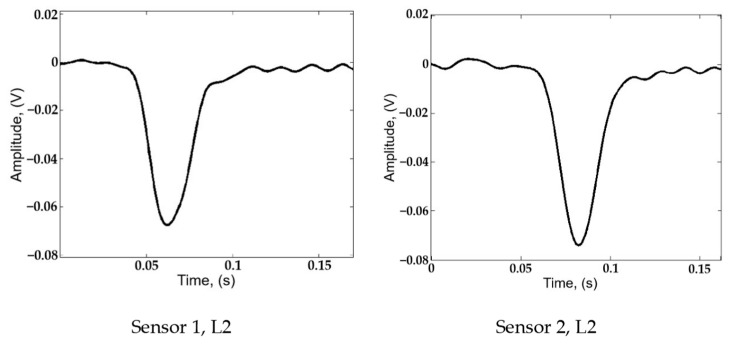
Typical responses of PZT sensor to NPW.

As the air in the pipeline escapes through the leakage point, a decrease in internal pressure will happen near the leakage point. The pipeline content both downstream and upstream moves towards the leakage point. The air flow in the pipeline then generates a negative pressure wave (NPW) propagating towards both sides of the pipeline from the leakage point. Following the decrease in internal pipeline pressure, the contraction of the pipe’s circumference causes strain variation on the pipe wall, which can be detected by the piezoelectric sensors bonded on the external surface of the pipeline. As a result, the signal waveforms are pulse-like, as shown in Figure 3, with Sensor 1 and Sensor 2 corresponding to the locations depicted in Figure 2.

More specifically, in Figure 3, when the pipe is undamaged, the sensor output remains at 0 V, as the internal pressure is stable. Then, the NPW reaches each specific sensor, and a downward, pulse-like signal emerges. The initial decline in the pulse reflects the drop in internal pressure as the NPW reaches the PZT sensor, while the subsequent rise shows the pressure stabilizing at a new baseline level. At the same time, the negative peak of the waveform indicates that the NPW is passing through the PZT sensor location, and two of the PZT sensors can be designated for use at the location of the leakage point.

## 4. Adaptive Grid Generation

### 4.1. The Signal Adjustment

The NPW velocity of u = 285.75 ± 23.6 m/s was measured in the same pipeline system [62], which is close to the theoretical 300 m/s NPW velocity reported in [63]. Therefore, this value was chosen, as it is situated in the upper margin of velocity estimation and for the convenience of calculations [61]. Meanwhile, the NPW’s propagation time from $r_{x}$ to $r_{y}$ can be calculated using the following equation:

(14) $$t=\left|r_{y}-r_{x}\right|/u$$

**Figure 4:**
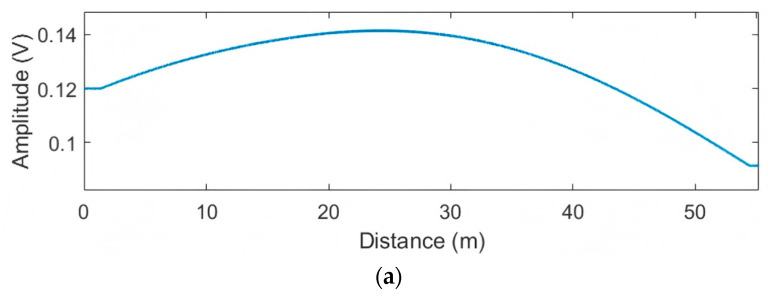

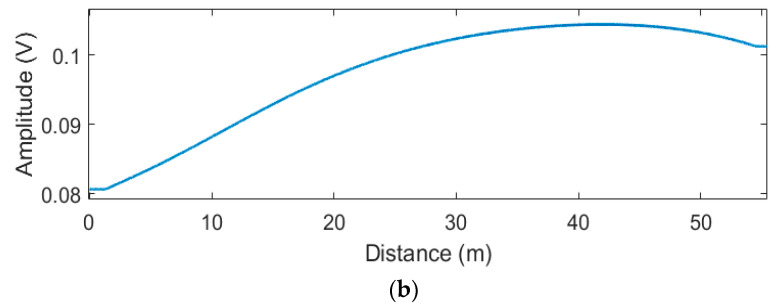
The maximum energy distribution curves based on the adjusted signals with the derived p values. (**a**) L2; (**b**) L4.

To reduce the localization resolution, the NPW signals captured by the two PZT sensors are adjusted via step a1. The resolution adjustment parameter p is induced as an unknown parameter. After deriving parameter p through step a2, the final p values corresponding to the four leakages are 0.5348, 0.5420, 0.5525, and 0.5485, respectively. The derived p value-based energy distribution curves plotted via Equation (12) are shown in Figure 4 (only the results for leaks L2 and L4 are shown due to space limitation). As illustrated in [48], the resolution increases when the parameter p is larger than 1. In contrast, the resolution will be decreased when the parameter p is less than 1. All the aforementioned parameter p values are around 0.5. Hence, by using the aforementioned parameter p values, the localization resolution is lower than that based on the original acquired NPW signals (normally, the localization resolution based on the original acquired NPW signal is equal to that with p=1). The leakage area revealed by using the aforementioned p values can cover a larger space. As shown in Figure 4, the −3 dB ranges of the results fill the entire monitoring area, ensuring that the localization leakage area can be covered by the entire monitoring region (as demonstrated in steps a1 and a2). Hence, the signal at the leakage point is not overlooked during the initial coarse grid meshing, which is the prerequisite for adaptive grid generation.

### 4.2. The Adaptive Grid

**Figure 5:**
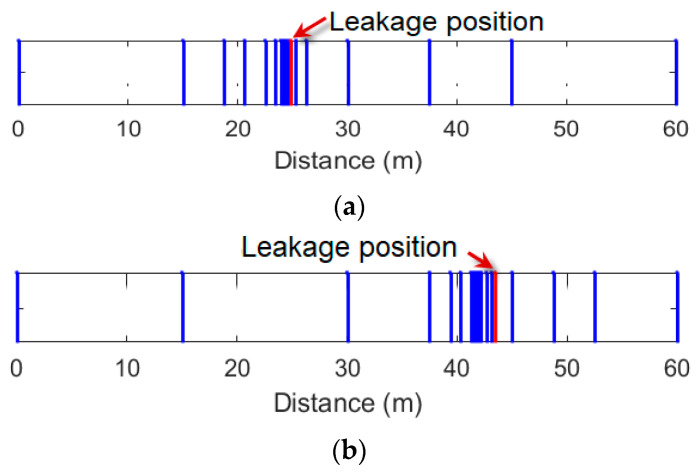
The grid distribution maps based on the proposed approach. (**a**) L2; (**b**) L4.

Since the low-resolution curves have local monotonicity and the peak is at the leakage points, the monitoring area can be meshed via steps b1–b6. The length of the initial monitoring area is 60 m, and the length of the initial grid is set as one half of the length of the monitoring area. The adaptive grid distribution maps are shown in Figure 5. In contrast to the fact that the non-leakage areas only contain sparse grids, most grids are concentrated in the leakage areas. Hence, the total number of grids is reduced significantly.

## 5. Leakage Localization Results and Comparison

### 5.1. Leak Localization Results

**Figure 6:**
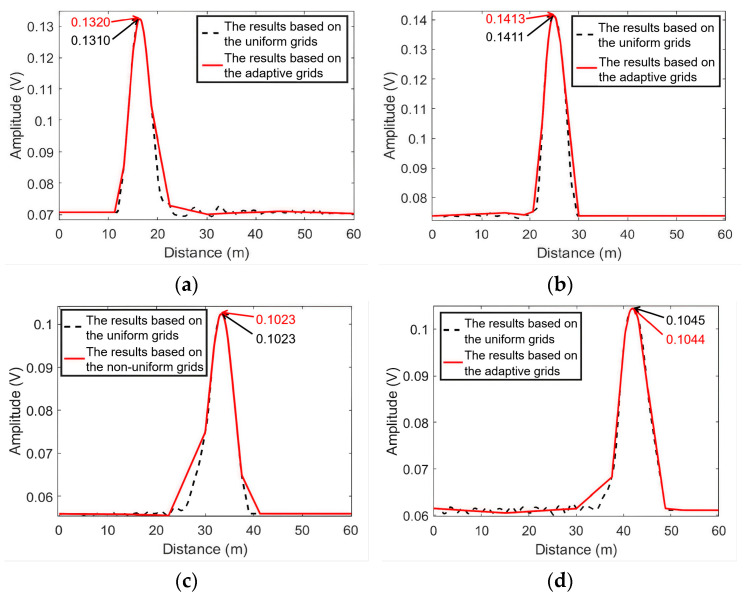
The results of the conventional TR localization method based on the uniform grids and the adaptive grids. (**a**) L1; (**b**) L2; (**c**) L3; (**d**) L4.

To compare our proposed approach with the uniform grid approach, the localization results based on uniform grids are also shown in this section. Since the minimum distance of the leakage position is 0.01 m, the grid step length of the uniform grid distribution is set to 0.01 m. The conventional time-reversal (TR) localization method in [35] is applied to compute the time-reversed signal energy distribution at the uniform grids and the adaptive grids. As shown in Figure 6, the results based on the adaptive grids are similar to those based on the uniform grids. In the non-leakage area, there is an apparent difference between the two curves, since the number of the adaptive grids in that area is small. However, in the leakage area, due to the densely distributed adaptive grids, the curves based on the two kinds of grids overlap each other. As a result, the leakage area can be successfully revealed by using the adaptive grids. The leak localization errors will be discussed later in Section 6.3.

### 5.2. Comparison of Leak Localization Cost

In order to compare the localization computation cost based on the adaptive grids with that based on the uniform grids, the number of uniform grids, the number of adaptive grids, and the computational time consumption based on the two kinds of grids are listed in Table 5 and Table 6. The comparison of two types of grids can be seen in Table 7.

As aforementioned, the localization computation using the adaptive grids contains the signal adjustment computation corresponding to steps a1–a2, the adaptive grid generation computation corresponding to steps b1–b6, and the conventional TR localization computation based on the adaptive grids. Therefore, the localization time consumption based on the adaptive grids will be separated into three parts—signal adjustment time, adaptive grid generation time, and conventional TR localization time—as shown in Table 6. The signal adjustment time consumption is about 1.1 s, the adaptive grid generation time consumption is about 0.45 s, and the conventional TR localization time consumption using the adaptive grids is about 0.45 s.

As can be seen in Table 7, it takes at least 87.759 s with 5581 uniform grids for the conventional TR localization method using uniform grids to localize the leakage. In contrast, merely 33 grids are needed for monitoring an area of 60 m length using the grids based on the proposed method. Furthermore, the total localization time consumption based on the adaptive grids is about 2 s, indicating that the computation time based on the adaptive grids is decreased to about 2.4% of that when using the uniform grids. Obviously, the adaptive grid generation approach can effectively decrease the cost of computing, speed up the localization, and give an accurate localization result.

## 6. Discussion

### 6.1. Discussion About Initial Grid Size

One key parameter of the proposed method is the initial grid size. The estimated locations of the four leakages based on the adaptive grid distribution with various initial grid sizes are listed in Table 8 and Table 9. Furthermore, for each test, the initial pipeline pressure is 20 psi.

The initial grid sizes in Table 8 and Table 9 are one-half and one-third of the monitoring area size, respectively. The positions of the initial grids vary with the change in the initial grid size, and the grid distribution changes with each iteration. Therefore, for four leakages, the maximum deviation is about 2% over 55.8 m.

The costs of localization computation using various initial grid sizes are listed in Table 6 and Table 10, respectively. The signal adjustment time consumption is related to the size of the initial monitoring area. The computation cost keeps basically the same in all cases due to having the same initial monitoring areas. The other two computation costs are mainly influenced by the number of adaptive grids. The fewer adaptive grids are employed, the lower the other two computation costs are. For Leakages 1 and 3, the change in the initial grid size changes the distribution of the adaptive grids, meaning that more adaptive grids are needed to find the leakage locations. Therefore, the adaptive grid generation costs increase. Additionally, the localization algorithm needs to process more grids; hence, the conventional TR localization time consumption based on the adaptive grids is also increased.

### 6.2. Discussion About the Ratio of the New Grid Size to the Previous Grid Size

Another key parameter of the proposed method is the size ratio of the new grid size to the previous grid size (denoted by β). In the proposed method, β is 0.5. For investigating the influence of this ratio, the localization results with β equal to 1/3 are listed in Table 11 and analyzed in the following section. Four leakage points are measured and calculated, as aforementioned.

The adaptive grid distribution becomes denser with the decrease in the ratio. The change in the adaptive grid distribution causes the deviation of the estimated leakage location, since the estimated leakage location depends on the adaptive grids’ positions. As listed in Table 11, the value of β decreases by 1/6, bringing about a maximum location change of 0.9 m. This is because the estimated leakage location is determined by selecting the grid point with the highest energy, and even small changes in grid spacing can lead to a slight shift in the identified peak. Nevertheless, the leakage can still be localized via finding a grid with the maximum signal energy. Therefore, the proposed approach can reveal the leakage positions under various β.

The localization computation costs based on various β are listed in Table 12. The signal adjustment time consumption is still about 1 s due to having the same initial monitoring area. β mainly influences the other two computation costs, namely the adaptive grid generation time and the localization time. A smaller new grid size means that more grids are required to fully cover the leakage area. Therefore, the time consumption for adaptive grid generation and leakage localization is increased to process more adaptive grids.

Generally speaking, the initial grid size and the size ratio have an impact on the computation cost based on the proposed method. However, the localization computation cost based on the proposed method is still much superior to that using the uniform grids.

### 6.3. Analysis of Localization Error

As shown in Table 8, Table 9 and Table 11, the maximum error based on a 55.8 m area is approximately 4%. The estimated localization errors are results of the following factors: First of all, in the model pipeline, the duration of the NPW signal is 0.05 s, as shown in Figure 3. The NPW travels a distance of 15 m or so in the duration, since the NPW velocity u equals 285.75 ± 23.6 m/s. As the distance between the ends of the pipeline and both the PZT sensors is less than 7.5 m, namely half of the NPW propagation distance, the superposition of the incident and reflected NPW postpones the peak of the NPW signal. Consequently, the estimated leak locations shift from the actual locations slightly. Furthermore, due to the dispersion effect, the peak of the signal shifts, and the duration of the signal is lengthened. Hence, the moments corresponding to the −3 dB values of the captured signals are also changed. This change leads to a slight calculation error of resolution adjustment parameter p, which can cause deviation in the localization results. Moreover, as the fact that the estimated leakage locations are located within the grids containing maximum signal energy, in essence, the distribution of adaptive grids, which causes different grid positions, is another factor of the localization error. As aforementioned, two parameters, namely initial grid size and size ratio, have an influence on the distribution of adaptive grids. Therefore, these two parameters also lead to localization errors. It is worthwhile to mention that the proposed method successfully reveals the leakage via finding the location with the maximum signal energy. Consequently, the estimated localization error is less.

The NPW, which can be detected by PZT patches bonded on the external surface of a pipeline, is generated from the circumferential contraction of a pipe. Thus, a much higher pressure, e.g., 1000 psi, is required for a steel pipeline with a pipe wall thickness of ¼ inch to generate detectable NPWs. Due to the limitations of our experimental conditions, it is difficult to pressurize metal pipelines to a high enough pressure in our laboratory. Therefore, in this paper, a PVC pipeline is applied for investigating the performance of the proposed method. In future, to improve the applicability of the proposed method, NPW propagation in pipes made from different materials and the influences of structural features, such as flanges, welding, and other joints, will be investigated. Additionally, challenges such as optimizing sensor layout density for comprehensive coverage and meeting real-time computational requirements in large-scale or complex pipeline networks should also be considered in actual deployment in line with the practical engineering scenario. Overall, the proposed method and adaptive sensor placement strategies with integrated edge computing frameworks will be optimized accordingly in the future by propagating NPWs along more complex pipeline networks (such as multi-branched, flange-connected networks, etc.) for the purpose of achieving rapid leakage localization in practical engineering applications.

## 7. Conclusions

Although the conventional time-reversal (TR) localization method can achieve high accuracy in pipeline leak detection, its practical application is limited due to the excessive computational load and high time consumption caused by the great number of grid points. In this study, a novel TR localization method based on an adaptive grid generation approach is developed to significantly reduce the number of required grids. Experimental validation on a 55.8 m PVC pipeline demonstrated that the proposed method successfully localizes leakages with only 33 adaptive grids, compared to the 5581 uniform grids required by conventional methods. As a result, the TR localization method based on adaptive grid generation consumes only 2.4% of the time of the conventional one. Overall, the proposed method offers a robust and computationally efficient solution for real-time pipeline leak detection, thereby facilitating its wider application in practical monitoring systems.

## Figures and Tables

**Table 1:** Table of the main notations.

Parameters	Symbol
$r_{L}$	The locations of the leakage
p	The resolution adjustment parameter
⊗	Convolution operation
$a_{n,L,m}$	Attenuation coefficient
$t_{n,L,m}$	NPW propagation time from $r_{L}$ to $r_{n}$
m	Forward propagation fields measured via the experiment
$f_{12}(t)$	Adjusted time function
$\delta \left(t-t_{n,L,m}\right)$	Ideal impulse
$I_{o}(r_{k})$	The localization functional value function
$v_{o}(r_{k})$	The maximum energy distribution value function
$s(r_{L},t)$	Negative pressure wave signal at leakage $r_{L}$
$x(r_{n},r_{L},t)$	Sensor-captured signal from sensor n to $r_{n}$
$h_{c}(r_{n},r_{k},t)$	The localization background functions
$g(r_{1},r_{k},t)$	The starting point of the monitoring area
$g(r_{2},r_{k},t)$	The end point of the monitoring area

**Table 2:** Leakage location.

Leakage Valve	Leakage 1 (L1)	Leakage 2 (L2)	Leakage 3 (L3)	Leakage 4 (L4)
Location from inlet (m)	15.55	24.84	34.21	43.47

**Table 3:** Sensor location.

PZT Sensors	Sensor 1	Sensor 2
Location from inlet (m)	1.32	54.46

**Table 4:** Parameters of the PZT material.

Parameters	Value
Relative Dielectric Constant	1900
Electromechanical Coupling Factor	0.72 (k33)
Piezoelectric Charge Constant (10 − 12 C/N or 10 − 12 m/V)	400 (d33)
Piezoelectric Voltage Constant (10 − 3 Vm/N or 10 − 3 m^2^/C)	24.8 (g33)

**Table 5:** The computation details of the conventional TR localization method based on the uniform grids.

Leakage	Grid Size	Conventional TR Localization Time	Number of Grids
L1	0.01 m	89.944 s	5581
L2	0.01 m	88.384 s	5581
L3	0.01 m	89.487 s	5581
L4	0.01 m	87.759 s	5581

**Table 6:** The computation details of the conventional TR localization method based on the adaptive grids (the initial grid size = ½ the monitoring area size and the new grid size = ½ the previous grid size).

Leakage	L1	L2	L3	L4
Signal adjustment time	1.093 s	1.161 s	1.205 s	1.154 s
Adaptive grid generation time	0.51 s	0.357 s	0.464 s	0.362 s
Conventional TR localization time	0.505 s	0.355 s	0.5 s	0.362 s
Total time	2.108 s	1.873 s	2.169 s	1.878 s
Number of grids	33	21	30	21

**Table 7:** Comparison of computation details between the two types of grids shown in Table 5 and Table 6.

Grids Type	Uniform Grids	Grids Based on Proposed Method
Maximum total computational time	89.944 s	2.169 s
Minimum total computational time	87.759 s	1.873 s
Average total computational time	88.894 s	2.007 s
Number of grids	All 5581	21–33

**Table 8:** The 5 repeated localization results based on the adaptive grids, where the initial grid size = ½ the monitoring area size and the new grid size = ½ the previous grid size.

Test	Leakage 1 (15.55 m)	Leakage 2 (24.84 m)	Leakage 3 (34.21 m)	Leakage 4 (43.47 m)
Result#1	16.41 m	24.38 m	33.75 m	41.25 m
Result#2	16.87 m	24.38 m	33.75 m	41.25 m
Result#3	16.35 m	24.38 m	33.28 m	41.25 m
Result#4	16.05 m	24.38 m	33.28 m	41.25 m
Result#5	16.87 m	24.38 m	33.75 m	41.25 m

**Table 9:** The 5 repeated localization results based on the adaptive grids, where the initial grid size = ⅓ the monitoring area size and the new grid size = ½ the previous grid size.

Test	Leakage 1 (15.55 m)	Leakage 2 (24.84 m)	Leakage 3 (34.21 m)	Leakage 4 (43.47 m)
Result#1	16.25 m	25 m	33.75 m	42.5 m
Result#2	16.25 m	25 m	33.75 m	41.9 m
Result#3	16.33 m	25 m	33.13 m	42.5 m
Result#4	16.09 m	25 m	33.13 m	42.5 m
Result#5	16.25 m	25 m	33.75 m	42.5 m

**Table 10:** The computation details of the conventional TR localization method based on the adaptive grids (the initial grid size = ⅓ the monitoring area size and the new grid size = ½ the previous grid size).

Leakage	L1	L2	L3	L4
Signal adjustment time	1.125 s	1.234 s	1.277 s	1.287 s
Adaptive grid generation time	0.366 s	0.391 s	0.398 s	0.382 s
Conventional TR localization time	0.325 s	0.417 s	0.325 s	0.368 s

**Table 11:** The 5 repeated localization results based on adaptive grids, where the initial grid size = ½ the monitoring area size and the new grid size = ⅓ the previous grid size.

Test	Leakage 1 (15.55 m)	Leakage 2 (24.84 m)	Leakage 3 (34.21 m)	Leakage 4 (43.47 m)
Result#1	16.67 m	24.88 m	33.33 m	41.67 m
Result#2	16.67 m	25 m	33.33 m	41.85 m
Result#3	16.36 m	25 m	33.33 m	41.67 m
Result#4	16.30 m	25 m	33.33 m	41.67 m
Result#5	16.67 m	25 m	33.33 m	41.67 m

**Table 12:** The computation details of the conventional TR localization method based on the adaptive grids (the initial grid size = ½ the monitoring area size and the new grid size = ⅓ the previous grid size).

Leakage	L1	L2	L3	L4
Signal adjustment time	1.082 s	1.154 s	1.158 s	1.240 s
Adaptive grid generation time	0.426 s	0.411 s	0.364 s	0.411 s
Conventional TR localization time	0.442 s	0.436 s	0.379 s	0.429 s

## Data Availability

The original contributions presented in the study are included in the article; further inquiries can be directed to the corresponding author.

## Abbreviations

The following abbreviations are used in this manuscript:

CS	Compressive Sensing
NPW	Negative Pressure Wave
PZT	Lead Zirconate Titanate
PVC	Polyvinyl Chloride
SHM	Structural Health Monitoring
TR	Time Reversal
VMD	Variational Mode Decomposition
